# Determining optimal needle size for decompression of tension pneumothorax in children – a CT-based study

**DOI:** 10.1186/s13049-019-0671-x

**Published:** 2019-10-11

**Authors:** Georg Leonhard, Daniel Overhoff, Lucas Wessel, Tim Viergutz, Marcus Rudolph, Michael Schöler, Holger Haubenreisser, Tom Terboven

**Affiliations:** 1 Department of Anaesthesiology and Intensive Care Medicine, University Medical Center Mannheim, Theodor-Kutzer-Ufer 1-3, 68167 Mannheim, Germany; 2 Institute of Clinical Radiology and Nuclear Medicine, University Medical Center Mannheim, Theodor-Kutzer-Ufer 1-3, 68167 Mannheim, Germany; 3 Department of Paediatric Surgery, University Medical Center Mannheim, Theodor-Kutzer-Ufer 1-3, 68167 Mannheim, Germany; 4 DRF Stiftung Luftrettung gemeinnützige AG, Filderstadt, Germany

**Keywords:** Tension pneumothorax, Children, Decompression, Needle size

## Abstract

**Background:**

For neonates and children requiring decompression of tension pneumothorax, specific recommendations for the choice of needle type and size are missing. The aim of this retrospective study was to determine optimal length and diameter of needles for decompression of tension pneumothorax in paediatric patients.

**Methods:**

Utilizing computed tomography, we determined optimal length and diameter of needles to enable successful decompression and at the same time minimize risk of injury to intrathoracic structures and the intercostal vessels and nerve. Preexisting computed tomography scans of the chest were reviewed in children aged 0, 5 and 10 years. Chest wall thickness and width of the intercostal space were measured at the 4th intercostal space at the anterior axillary line (AAL) on both sides of the thorax. In each age group, three needles different in bore and length were evaluated regarding sufficient length for decompression and risk of injury to intrathoracic organs and the intercostal vessels and nerve.

**Results:**

197 CT-scans were reviewed, of which 58 were excluded, resulting in a study population of 139 children and 278 measurements. Width of the intercostal space was small at 4th ICS AAL (0 years: 0.44 ± 0.13 cm; 5 years: 0.78 ± 0.22 cm; 10 years: 1.12 ± 0.36 cm). The ratio of decompression failure to risk of injury at 4th ICS AAL was most favourable for a 22G/2.5 cm catheter in infants (Decompression failure: right: 2%, left: 4%, Risk of injury: right: 14%, left: 24%), a 22G/2.5 cm or a 20G/3.2 cm catheter in 5-year-old children (20G/3.2 cm: Decompression failure: right: 2.1%, left: 0%, Risk of injury: right: 2.1%, left: 17%) and a 18G/4.5 cm needle in 10-year-old children (Decompression failure: right: 9.5%, left: 9.5%, Risk of injury: right: 7.1%, left: 11.9%).

**Conclusions:**

In children aged 0, 5 and 10 years presenting with a tension pneumothorax, we recommend 22G/2.5 cm, 20G/3.2 cm and 18G/4.5 cm needles, respectively, for acute decompression.

## Background

Tension pneumothorax is a rare but rapidly fatal condition in paediatric patients. Contrary to a growing body of data for adult patients [1–3], little is known about the required depth of puncture and the risk of injury to underlying vital structures in children. Mandt et al. were the first to review chest CTs of children < 13 years of age to measure chest wall thickness (CWT) [4]. We recently reported the results of a CT-based study evaluating depth to intrathoracic vital structures in children in this journal [5]. Due to the high risk of injury to intrathoracic vital structures at 2nd ICS MCL, from our point of view the 4th ICS AAL represents the insertion site of choice outside the context of Point-of-Care Ultrasound. The optimal needle should be long enough for successful pleural decompression, but short enough to minimize the risk of injury to vital intrathoracic structures. Furthermore, the diameter of the needle should be small enough to avoid injury of the intercostal vessels in the small intercostal spaces encountered in paediatric patients. In this CT-based investigation, we sought to determine the optimal length and diameter of the needle for decompression at the 4th ICS AAL in children aged 0, 5 and 10 years.

## Methods

All data were collected retrospectively from February to May 2018. In order to detect age-related changes in the recorded parameters whilst maintaining clarity about the results, it was decided to evaluate children from three different age groups. After collecting 197 preexisting thoracic CT images of children aged 0, 5 and 10 years from the local picture archiving and communications system, 58 patients were excluded because of various pathologies making the required measurements impossible or inaccurate (Additional file 1: Table S1). The most frequent indications for imaging in the remaining children were oncologic staging/searching for metastases, trauma and suspected pulmonary diseases.

### Data acquisition

All thoracic CT examinations were performed on Siemens Somatom Definition Flash (2nd generation DSCT) or Siemens Emotion 16 (16 slice MSCT, both Siemens Healthineers, Forchheim, Germany) CT scanners, with slice thickness 1.5 mm and increment 1.0 mm. Images were reconstructed using both lung and soft tissue reconstruction kernels to allow an optimal visualization of all structures and imported to a PACS Workstation (Aycan OsiriX PRO v.2.10, Aycan Digitalsysteme GmbH, Würzburg, Germany). Analysis of all images was performed in multiplanar reconstructions by a specialist in paediatric radiology.

### Measurements

**Fig. 1:**
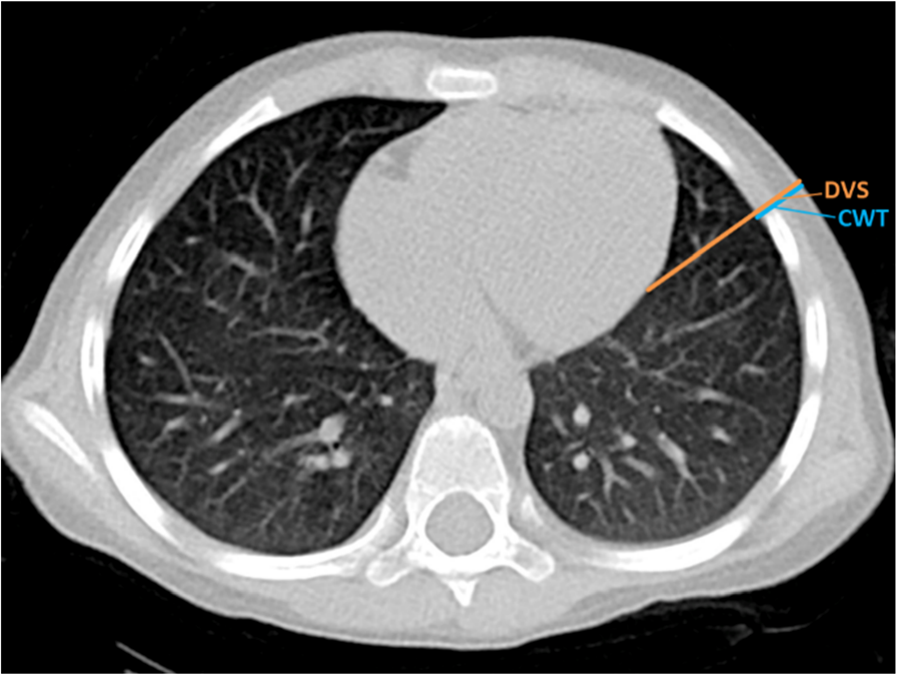
Graphical display of measurements. 4th ICS AAL. DVS: depth to vital structure, CWT: chest wall thickness

**Fig. 2:**
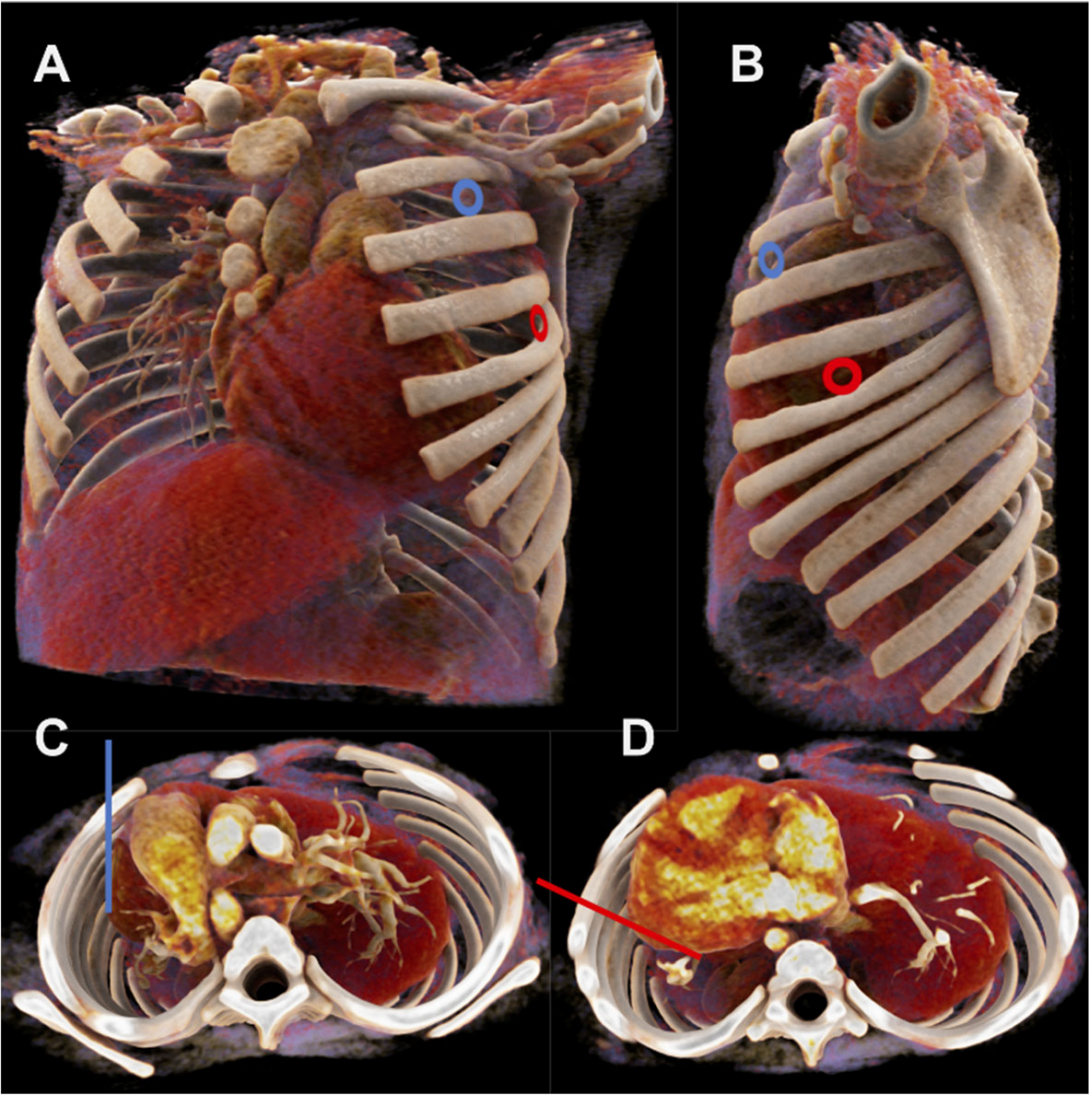
Possible insertion sites and intrathoracic structures. Reconstruction of a thoracic CT of a 21 months old girl. **a** and **b**: Insertion sites at 2nd ICS MCL and 4th ICS AAL. **c**: 2nd ICS midclavicular line, sagittal insertion. View from apical. **d**: 4th ICS anterior axillary line, insertion perpendicular to the chest wall. View from apical

Measurements were performed on both sides of the thorax at the 4th ICS AAL. A detailed description of the measurements and definitions is shown in Table 1 and Fig. 1. The outer diameter and length of the investigated needles are shown in Table 2. Figure 2 shows possible insertion sites for needle decompression and the proximity to intrathoracic vital structures.

**Table 1:** Description of measurements

Width of the ICS	Bottom edge of the 4th rib to the top edge of the 5th rib, measured in the coronar plane.
Difference between width of the ICS and outer diameter of the needles	(Width of the ICS in cm) – (outer diameter of the respective needle in cm).
Chest wall thickness	Distance from the skin surface to the pleural cavity, measured perpendicular to the chest wall in the axial plane.
Depth to vital structure	Distance from the skin surface to the intersection with the closest vital structure in the direction of puncture. Measured in the axial plane.
Decompression failure	Chest wall thickness > length of the needle
Risk of injury	Depth to vital structure < length of the needle
Vital structures	Pericardium, aorta, vena cava inferior and superior, pulmonary vessels including larger intraparenchymal branches, thymus gland.

**Table 2:** Outer diameter and length of the investigated catheters (BD Venflon™ Pro Safety)

	Outer Diameter	Length
24G	0.07 cm	1.9 cm
22G	0.09 cm	2.5 cm
20G	0.11 cm	3.2 cm
18G	0.13 cm	4.5 cm
16G	0.18 cm	4.5 cm

### Statistical analysis

Measurement data were imported to JMP 13.0 (SAS Institute Inc., Cary, NC) for statistical analysis. The Mann-Whitney U test was used to compare continuous variables (hereinafter presented as mean ± standard deviation), with statistical significance being set at *p* ≤ 0.05.

## Results

Fifty infants, 47 5-year-olds and 42 10-year-olds were included in the final analysis. The respective mean ages were 0.42 (±0.32) years, 5.49 (±0.28) years and 10.45 (±0.30) years.

### 0-year-old children

#### Width of the ICS

**Fig. 3:**
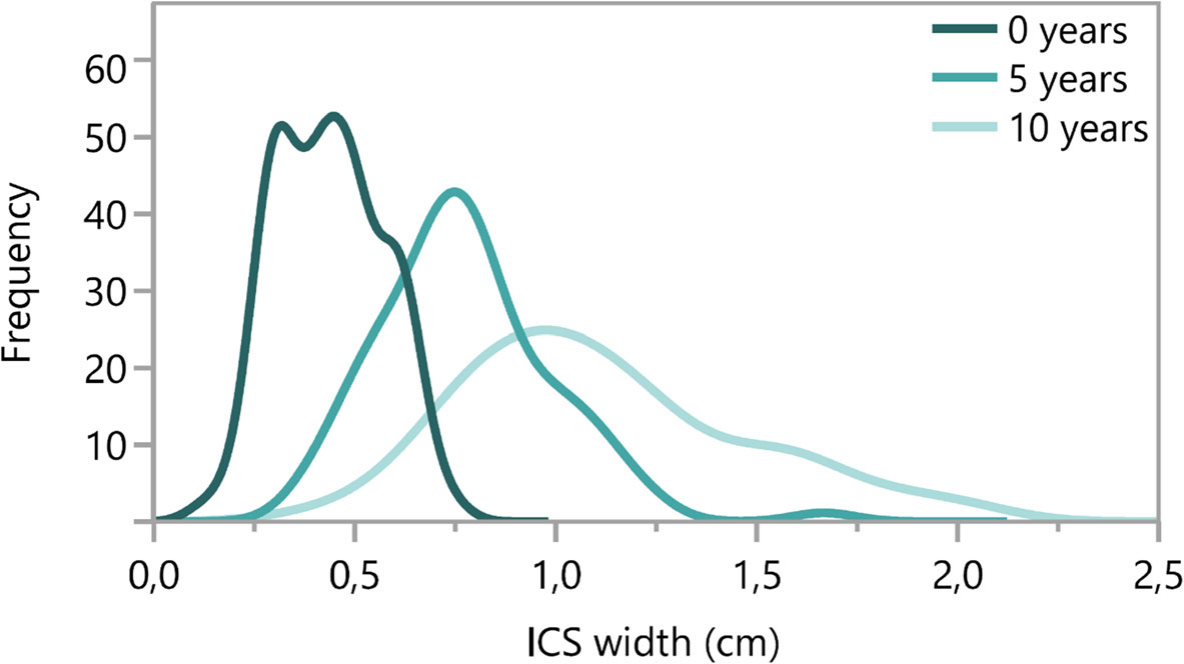
Width of the ICS in 0, 5 and 10-year-old children. Density curves for 4th ICS AAL

The mean width of the 4th ICS AAL was 0.44 ± 0.13 cm (Minimum: 0.13 cm, Maximum: 0.75 cm). Figure 3 demonstrates the density curves for width of the ICS in the three age groups. A high proportion of infants presented with a width of the ICS smaller than 0.25 cm. Table 3 shows a calculation of the difference between width of the ICS and the outer diameter of needles investigated in this study.

**Table 3:** Difference between the width of the ICS and the diameter of different needles for 0-year-old children, mean ± standard deviation and minimum, all measurements in cm

Diameter		Length	4. ICS AAL	
			Mean (SD)	Minimum
24G	0.07 cm	1.9 cm	0.37 ± 0.13	0.06
22G	0.09 cm	2.5 cm	0.35 ± 0.13	0.04
20G	0.11 cm	3.2 cm	0.33 ± 0.13	0.02

#### Decompression failure and risk of injury to intrathoracic structures

At 4th ICS AAL decompression with a 20G cannula would not have failed in any case on both sides of the thorax, but is accompanied by a high risk of injury at full insertion of the needle (left: 62%, right: 18%). Using a shorter 22G catheter only marginally increases failure rate (left: 4%, right: 2%), but especially in the left hemithorax dramatically reduces the rate of possible injury (left: 24%, right: 14%). 24G catheters appear relatively safe regarding injuries, but are too short for decompression in a large proportion of infants (left: 20%, right: 18%). Results are shown in Table 4.

**Table 4:** Decompression failure and risk of injury in 0-year-old children

4th Intercostal Space, Anterior Axillary Line						
Needle Size	Diameter	Length	4. ICS AAL right		4. ICS AAL left	
			Decompression failure	Risk of injury	Decompression failure	Risk of injury
24G	0.07 cm	1.9 cm	18.0%	0.0%	20.0%	6.0%
22G	0.09 cm	2.5 cm	2.0%	14.0%	4.0%	24.0%
20G	0.11 cm	3.2 cm	0.0%	18.0%	0.0%	62.0%

#### 5-year-old children

Mean width of the 4th ICS at AAL was 0.78 ± 0.22 cm. The mean differences between width of the ICS and the outer diameter of the needles were 0.65–0.69 cm. The minimum differences in this age group ranged from 0.24–0.28 cm for the investigated 18G, 20G and 22G cannulas (Table 5). Figure 3 demonstrates the density curves for width of the ICS.

**Table 5:** Difference between the width of the ICS and the diameter of different needles for 5-year-old children, mean ± standard deviation and minimum, all measurements in cm

Diameter		Length	4. ICS AAL	
			Mean (SD)	Minimum
24G	0.09 cm	2.5 cm	0.69 ± 0.22	0.28
22G	0.11 cm	3.2 cm	0.67 ± 0.22	0.26
20G	0.13 cm	4.5 cm	0.65 ± 0.22	0.24

#### Decompression failure and risk of injury to intrathoracic structures

Rate of decompression failure is low for all investigated needles at 4th ICS AAL (0-2.1%). Risk of injury is relatively low for a 22G needle (right: 2.1%, left 4.3%) but considerably rises especially on the left hemithorax for longer needles (left hemithorax: 20G: 17%; 18G: 59.6%; right hemithorax: 20G: 2.1%; 18G: 21.3%). Results are shown in Table 6.

**Table 6:** Decompression failure and risk of injury in 5-year-old children

4th Intercostal Space, Anterior Axillary Line						
Needle Size	Diameter	Length	4. ICS AAL right		4. ICS AAL left	
			Decompression failure	Risk of injury	Decompression failure	Risk of injury
22G	0.09 cm	2.5 cm	2.1%	2.1%	2.1%	4.3%
20G	0.11 cm	3.2 cm	2.1%	2.1%	0.0%	17.0%
18G	0.13 cm	4.5 cm	0.0%	21.3%	0.0%	59.6%

#### 10-year-old children

At 4th ICS AAL mean width of the ICS was 1.12 ± 0.36 cm (Minimum: 0.50 cm, Maximum: 2.05 cm). Figure 3 demonstrates the density curves for width of the ICS. Table 7 shows a calculation of the difference between width of the ICS and the outer diameter of needles investigated in this study.

**Table 7:** Difference between the width of the ICS and the diameter of different needles for 10-year-old children, mean ± standard deviation and minimum, all measurements in cm

Diameter		Length	4. ICS AAL	
			Mean (SD)	Minimum
20G	0.11 cm	3.2 cm	1.01 ± 0.36	0.27
18G	0.13 cm	4.5 cm	0.99 ± 0.36	0.25
16G	0.15 cm	4.5 cm	0.94 ± 0.36	0.2

#### Decompression failure and risk of injury to intrathoracic structures

Rate of decompression failure at 4th ICS AAL is 9.5% with 16G or 18G cannulas but rises to 21.4% with a 20G cannula. Risk of injury on the right hemithorax increases when using longer catheters (20G: 2.4%; 18/16G: 7.1%). On the left hemithorax risk of injury is higher than on the right, but comparable between all types of investigated needles (9.5-11.9%). Results are shown in Table 8.

**Table 8:** Decompression failure and risk of injury in 10-year-old children

4th Intercostal Space, Anterior Axillary Line						
Needle Size	Diameter	Length	4. ICS AAL right		4. ICS AAL left	
			Decompression failure	Risk of injury	Decompression failure	Risk of injury
20G	0.11 cm	3.2 cm	21.4%	2.4%	21.4%	9.5%
18G	0.13 cm	4.5 cm	9.5%	7.1%	9.5%	11.9%
16G	0.18 cm	4.5 cm	9.5%	7.1%	9.5%	11.9%

## Discussion

The incidence and therefore the exposure of staff to tension pneumothorax in children is low and providers often feel uncomfortable with and not well prepared for this situation [6–8]. The technique of choice for emergency decompression in children remains a matter of debate. Finger thoracostomy, as recommended by Vassallo et al. [9], requires some surgical skills due to the tiny intercostal spaces in children. Needle decompression appears to be a more simple, less invasive, easier to learn and easier to retain procedure [10]. Recommendations for needle decompression in adults are based on a growing body of evidence [1, 3]. Little evidence exists for paediatric patients and recommendations mostly seem to be transferred from the adult literature [11]. Mandt et al. were the first to review chest CTs of children < 13 years of age to measure chest wall thickness (CWT) [4]. The authors established 4 groups based on Broselow™ colour as determined by height. The reported median CWT of these age groups agrees quite well with the measurements made by our group (data shown in the Additional file 1: Table S2) [5]. Based on median CWT, Mandt et al. conclude that a 3.8 cm needle is of sufficient length to access the pleural cavity in most children. In the present study we evaluated different sizes of needles in regard to the rate of decompression failure and the risk of injury to intrathoracic structures at full insertion of the needle. We furthermore evaluated the risk of injury to the intercostal vessels and nerve by needles of different diameters.

Clemency et al. defined the acceptable theoretical success rate (catheter longer than CWT) for needle decompression at 95%, Hecker et al. at 90% [2, 12]. In our study the theoretical rate of success in 0-year-old children was ≥96% for 22G/2.5 cm and 100% for 20G/3.2 cm needles at the investigated insertion sites. In 5-year-old children 22G/2.5 cm needles are of sufficient length for decompression at 4th ICS AAL in 97.9%. In 10-year-old children 18G/4.5 cm catheters are needed for theoretical rates of decompression success > 90%. Due to the more cephalad position of the diaphragm in small children, the heart is in a more transverse position in the thoracic cavity. Therefore, in paediatric thoracic ultrasound investigations at 2nd ICS MCL, the heart can often be seen moving into the ultrasound window during expiration. Data from studies on localizing insertion sites for needle decompression in adults show that misplacement rates for 2nd ICS MCL were high and that the midclavicular line was located too medially by high proportion of participants [13, 14]. This further rises the risk of accidentally puncturing the heart or the thymus gland in children. Due to the devastating complications of injuring these intrathoracic organs we cannot recommend 2nd ICS MCL in 0- and 5-year-old children [5]. At 4th ICS AAL on the left hemithorax the heart is in close proximity to the insertion site and insertion of the needle has to be immediately stopped after entering the pleural cavity to avoid cardiac injury [5].

However, no vital structures were found directly adjacent to the thoracic wall (data shown in the Additional file 1: Tables S3-S5). 4th ICS AAL therefore represents the insertion site of choice and was investigated regarding the optimal size of the decompression needle in this study. Selection of the appropriate needle requires special attention because of the small width of the intercostal space. Laceration of the intercostal artery with massive haemorrhage is a complication that is described in adults [15] and seems even more likely in the narrow intercostal spaces in small children. In 1998, Roberts et al. described a rate of haemothorax of 2% after placing pigtail catheters for evacuation of pleural air or fluid in children [16]. Complications were significantly higher in children weighing less than 5 kg. In our study, mean width of the 4th ICS at AAL was only 0.44 ± 0.13 cm in infants. The minimum difference between the width of the ICS and the diameter of the needles at 4th ICS AAL in this age group ranged from 0.02 cm (20G) to 0.06 cm (24G). To avoid injury of the intercostal vessels we therefore strongly recommend not to choose large bore cannulas and pay particular attention to an insertion of the needle just superior to the upper edge of the lower rib. A problem with smaller bore cannulas is reduced airflow, possibly impeding evacuation of pneumothorax. Furthermore, obstruction of small bore needles due to debris or blood clots or dislocation of the needle can also easily lead to decompression failure. In case of high urgency, the use of a second cannula can also be considered. This problem could also be solved by placing a larger bore pigtail catheter in an over-the-wire technique after puncturing the thoracic cavity with a smaller needle. Noh et al. however reported on no significant difference of evacuation success in adult patients with pneumothorax when using 7 French compared to 12 French pigtail catheters [17]. In 5-year-old children the width of the ICS is clearly bigger, but minimum difference between the investigated needles (22G, 20G, 18G) and the width of the ICS at 4th ICS AAL still is around 0.25-0.3 cm. A slight deviation from correct angle of entry of a larger bore cannula can still easily injure the intercostal vessels in this age group. Even in 10-year-old children this difference stays disconcertingly small when using a 16G cannula (0.2 cm).

In trauma patients pre-hospital chest decompression often seems to be performed without a clear indication [18]. Needle decompression performed in children in absence of a pneumothorax carries a high risk of injury to intrathoracic organs in children. All these matters show the importance of choosing the right insertion site and the optimal needle regarding length and bore for decompression.

### Limitations

This study has several limitations. First of all, the presence of a pneumothorax most likely reduces the risk of injuring intrathoracic organs as the accumulated air in the pleural space provides an additional buffer zone. As measurements were taken in children without pneumothorax, the effects from displacement of intrathoracic structures on the risk of injury could not be investigated. Based on clinical examination alone, diagnosis of a pneumothorax however is difficult and faulty. Without the use of point of care ultrasound/radiography pneumothorax is probably over diagnosed in trauma patients. Regarding the thickness of the chest wall, compression of subcutaneous tissue by the tip of the needle cannot be assessed reliably in CT. Compression might lead to a reduction of required insertion depth for decompression. In contrast, subcutaneous emphysema might lead to an increased thickness of the chest wall and thus to decompression failure. Our study presupposes that puncture sites are correctly identified and puncture is performed perpendicularly to the chest wall at 4th ICS, whereas misplacement was found to be frequent in several studies [15, 16] and could be even more likely in the smaller spatial relationships in children. The effect of errors in identification of puncture site and angle of entry, especially on the risk of injury to underlying vital structures, is yet to be determined. Finally we only evaluated children aged 0, 5 and 10 years and cannot make any definitive statement on the generalizability of the date to other age groups.

## Conclusions

To reduce the risk of injury to the intercostal vessels and intrathoracic structures we recommend a 22G/2.5 cm needle for infants, a 20G/3.2 cm needle for 5 year-old-children and an 18G/4.5 cm needle for 10-year-old children. In small infants and newborns, the use of a 24G cannula should be considered. To avoid an unnecessarily deep needle penetration, puncture should be performed under guidance by aspiration of air via a syringe and needle movement should be immediately stopped after the aspiration of air. Due to the proximity of the heart to the insertion site, this is of particular importance on the left hemithorax. Whenever possible, ultrasound should be used for confirmation of a pneumothorax, to measure chest wall thickness and confirm lack of underlying vital structures (e.g. the heart) before puncture and hence minimize depth of needle insertion and reduce the risk of injuring vital structures.

## Supplementary information

**Additional file 1: Table S1.** Reasons for exclusion. **Table S2.** Chest wall thickness (cm), mean ± SD. **Table S3.** Structures directly adjacent to the thoracic wall, 0-year-old children. **Table S4.** Structures directly adjacent to the thoracic wall, 5-year-old children. **Table S5.** Structures directly adjacent to the thoracic wall, 10-year-old children.

## Abbreviations

AAL: Anterior axillary line
CT: Computed tomography
CWT: Chest wall thickness
DVS: Depth to vital structure
EMS: Emergency medicine service
ICS: Intercostal space
MCL: Medioclavicular line
SD: Standard deviation
