# Supplementary material for: Determining optimal needle size for decompression of tension pneumothorax in children – a CT-based study
Source: Scand J Trauma Resusc Emerg Med. 2019 Oct 11;27:90. doi: 10.1186/s13049-019-0671-x (PMC6788035; doi:10.1186/s13049-019-0671-x)
Supplement: Supplementary file 1 — Additional file 1: Table S1. Reasons for exclusion. Table S2. Chest wall thickness (cm), mean ± SD. Table S3. Structures directly adjacent to the thoracic wall, 0-year-old children. Table S4. Structures directly adjacent to the thoracic wall, 5-year-old children. Table S5. Structures directly adjacent to the thoracic wall, 10-year-old children. [file 13049_2019_671_MOESM1_ESM.docx]

**Supplementary Material**

**Table S1:** Reasons for exclusion

| **Reason for exclusion** | **n (0 years)** | **n (5 years)** | **n (10 years)** | **n (total)** |
| --- | --- | --- | --- | --- |
| Mediastinal shift | 12 | 1 | 1 | 14 |
| Pulmonary infiltration | 11 | 1 | 0 | 12 |
| Congenital pulmonary airway malformation | 8 | 0 | 0 | 8 |
| Pulmonary bullae | 6 | 0 | 0 | 6 |
| Pleural effusion/empyema | 3 | 2 | 0 | 5 |
| Poor image quality | 1 | 3 | 0 | 4 |
| Spinal misalignment | 1 | 1 | 1 | 3 |
| Emphysema | 3 | 0 | 0 | 3 |
| Congenital diaphragmatic hernia | 2 | 0 | 0 | 2 |
| Intrathoracic mass | 1 | 0 | 0 | 1 |
| **Total** | 48 | 8 | 2 | 58 |

**Table S2:** Chest wall thickness (cm), mean ±SD

|  | **0 years** | | **5 years** | | **10 years** | |
| --- | --- | --- | --- | --- | --- | --- |
|  | *right* | *left* | *right* | *left* | *right* | *left* |
| **2^nd^ ICS MCL** | 1.52 | 1.56 | 1.76 | 1.81 | 2.61 | 2.63 |
|  | [±0.51] | [±0.61] | [±0.48] | [±0.48] | [±1.15] | [±1.23] |
| **4^th^ ICS AAL** | 1.38 | 1.41 | 1.34 | 1.28 | 2.21 | 2.19 |
|  | [±0.48] | [±0.50] | [±0.46] | [±0.41] | [±1.33] | [±1.30] |
| **p** | 0.11 | 0.15 | <0.05 | <0.05 | <0.05 | <0.05 |

**Table S3:** Structures directly adjacent to the thoracic wall, 0-year-old children

|  | **Right hemithorax** | | **Left hemithorax** | |
| --- | --- | --- | --- | --- |
|  | 2^nd^ ICS MCL | 4^th^ ICS AAL | 2^nd^ ICS MCL | 4^th^ ICS AAL |
| None | 48 (96%) | 50 (100%) | 49 (98%) | 0 (0%) |
| Heart | 0 (0%) | 0 (0%) | 0 (0%) | 0 (0%) |
| Thymus gland | 2 (4%) | 0 (0%) | 1 (2%) | 0 (0%) |

**Table S4:** Structures directly adjacent to the thoracic wall, 5-year-old children

|  | **Right hemithorax** | | **Left hemithorax** | |
| --- | --- | --- | --- | --- |
|  | 2^nd^ ICS MCL | 4^th^ ICS AAL | 2^nd^ ICS MCL | 4^th^ ICS AAL |
| None | 47 (100%) | 47 (100%) | 46 (97.9%) | 47 (100%) |
| Heart | 0 (0%) | 0 (0%) | 1 (2.1%) | 0 (0%) |
| Thymus gland | 0 (0%) | 0 (0%) | 0 (0%) | 0 (0%) |

**Table S5:** Structures directly adjacent to the thoracic wall, 10-year-old children

|  | **Right hemithorax** | | **Left hemithorax** | |
| --- | --- | --- | --- | --- |
|  | 2^nd^ ICS MCL | 4^th^ ICS AAL | 2^nd^ ICS MCL | 4^th^ ICS AAL |
| None | 42 (100%) | 42 (100%) | 42 (100%) | 42 (100%) |
| Heart | 0 (0%) | 0 (0%) | 0 (0%) | 0 (0%) |
| Thymus gland | 0 (0%) | 0 (0%) | 0 (0%) | 0 (0%) |
